# Analysis of the ex-vivo transformation of semen, saliva and urine as they dry out using ATR-FTIR spectroscopy and chemometric approach

**DOI:** 10.1038/s41598-021-91009-5

**Published:** 2021-06-04

**Authors:** Tanurup Das, Abhimanyu Harshey, Ankit Srivastava, Kriti Nigam, Vijay Kumar Yadav, Kapil Sharma, Arun Sharma

**Affiliations:** 1 Dr. A.P.J. Abdul Kalam Institute of Forensic Science & Criminology, Bundelkhand University, Jhansi, 284128 Uttar Pradesh India; 2 DFS, Himachal Pradesh, Junga, Shimla, 171218 Himachal Pradesh India

**Keywords:** Biochemistry, Analytical chemistry, Biochemistry

## Abstract

The ex-vivo biochemical changes of different body fluids also referred as aging of fluids are potential marker for the estimation of Time since deposition. Infrared spectroscopy has great potential to reveal the biochemical changes in these fluids as previously reported by several researchers. The present study is focused to analyze the spectral changes in the ATR-FTIR spectra of three body fluids, commonly encountered in violent crimes i.e., semen, saliva, and urine as they dry out. The whole analytical timeline is divided into relatively slow phase I due to the major contribution of water and faster Phase II due to significant evaporation of water. Two spectral regions i.e., 3200–3400 cm^−1^ and 1600–1000 cm^−1^ are the major contributors to the spectra of these fluids. Several peaks in the spectral region between 1600 and 1000 cm^−1^ showed highly significant regression equation with a higher coefficient of determination values in Phase II in contrary to the slow passing Phase I. Principal component and Partial Least Square Regression analysis are the two chemometric tool used to estimate the time since deposition of the aforesaid fluids as they dry out. Additionally, this study potentially estimates the time since deposition of an offense from the aging of the body fluids at the early stages after its occurrence as well as works as the precursor for further studies on an extended timeframe.

## Introduction

Body fluids are recurrently confronted as major evidence in violent crimes^1^. The potential application of different body fluids typically ranging from their identification to the successful extraction of DNA and its profiling^2^. All of these fluids experience instant biochemical change as they come out from the body. This change can be referred as aging^1–4^. Some of these changes are rapid and the remaining are gradual^3^, but both the changes are significant to reveal one of the most important aspects of forensic examination; ‘time since the deposition’ of a crime^4^. The estimation of ‘time since deposition (TSD) of a body fluid at the crime scene potentially solves the problem of situating the time of an offense^3^. The TSD of body fluids additionally counter a wide range of issues regarding the crime scene investigation^3,4^. Most of the TSD studies are based on bloodstain aging^2–7^ except for a solitary publication on semen very recently^8^. All these studies except one^9^ investigated the TSD for an extended period, escaping the initial changes in the fluid. With the inception of the TSD estimation study in the early twentieth century, various researchers have investigated this phenomenon on bloodstains^3,4^. Since then the techniques used for TSD estimation went through a drastic evolutionary transformation. The analytical technology gradually transformed into nondestructive from destructive methods^3,4,10^. A variety of techniques including Gas chromatography^11^, Liquid chromatography^12–14^, Oxygen electrode^3,15^, microRNA based assay^3,16–19^, Color transformation chart^20^, Electron paramagnetic resonance^21–23^, Reflectance spectroscopy in Ultraviolet, Visible and Infrared (IR) region^24–26^, smartphone imaging^27^, IR absorption^1,3,4,8,9,28,29^, Raman scattering^4,6,7,11,30^ and fluorescence spectroscopy is explored around the world for the TSD estimation of blood (peripheral and menstrual)^31^ and semen^8^. As an age prediction tool, most of these methods realistically depict promising results. Yet, few methodological limitations hinder the universal acceptability of one or more of these techniques as the stand-alone tool for TSD studies except IR absorption and Raman scattering collectively known as vibrational spectroscopy^4,10,11^. Leading researchers in the forensic sciences community around the world are significantly attracted towards the vibrational spectroscopic technique due to its several advantages over others. It reliably provides a rapid non-destructive analysis of evidence in forensic investigation^4,10,11^. The last decade showed significant growth in the TSD estimation studies by using vibrational spectroscopic techniques^4^. Despite the equal importance of semen, saliva, and urine as evidence in crime scene investigations, blood always received special attention in the research community^3,4,10,11,32,33^. All the three aforesaid fluids including blood are consistently found in the broad range of homicides, suicides, rapes, and other sexual assaults^2,34,35^. Although, it is not very frequent that an investigating team can reach the crime scene as early as the fluid is still under drying process, but in several instances, investigators may come across a situation where the fluid is still drying. The earliest time after an offense has enormous importance as this is the time when most of the information in a crime scene remains unchanged and also during this timeframe, the most significant information can be obtained from postmortem. Additionally, the empirical relation between the initial spectral change and TSD can help an investigator to differentiate between two or more overlapped older and fresh stains^9^. Zhang et al.^9^ explored this specific and significant dimension of body fluid aging in 2016 and established a potential age estimation framework from the initial changes in the ATR-FTIR spectra of bloodstain collected from Sprague Dawley rats up to its drying under different conditions (temperature, humidity, and concentration) as the drying of fluid is affected by several factors like the component of the fluid, environmental conditions, and the supporting substrate^9,11,36^. The present study is carefully focused to analyze the initial infrared spectral changes of semen, saliva, and urine as they dry out and estimate the TSD based on the absorption changes in specific peaks. Since, Zha et al.^8^ experimented with the change of semen stains up to 1 week, in which the first spectra were acquired 12 hours after the deposition of semen ignoring the initial changes. Hence, semen is also included in the present study. In Attenuated total reflectance (ATR) Fourier transforms infrared (FTIR) spectroscopy is combined with the chemometric approach to investigate the spectral changes and estimate the TSD respectively.

## Results and discussion

### Spectral change over the drying period

**Figure 1:**
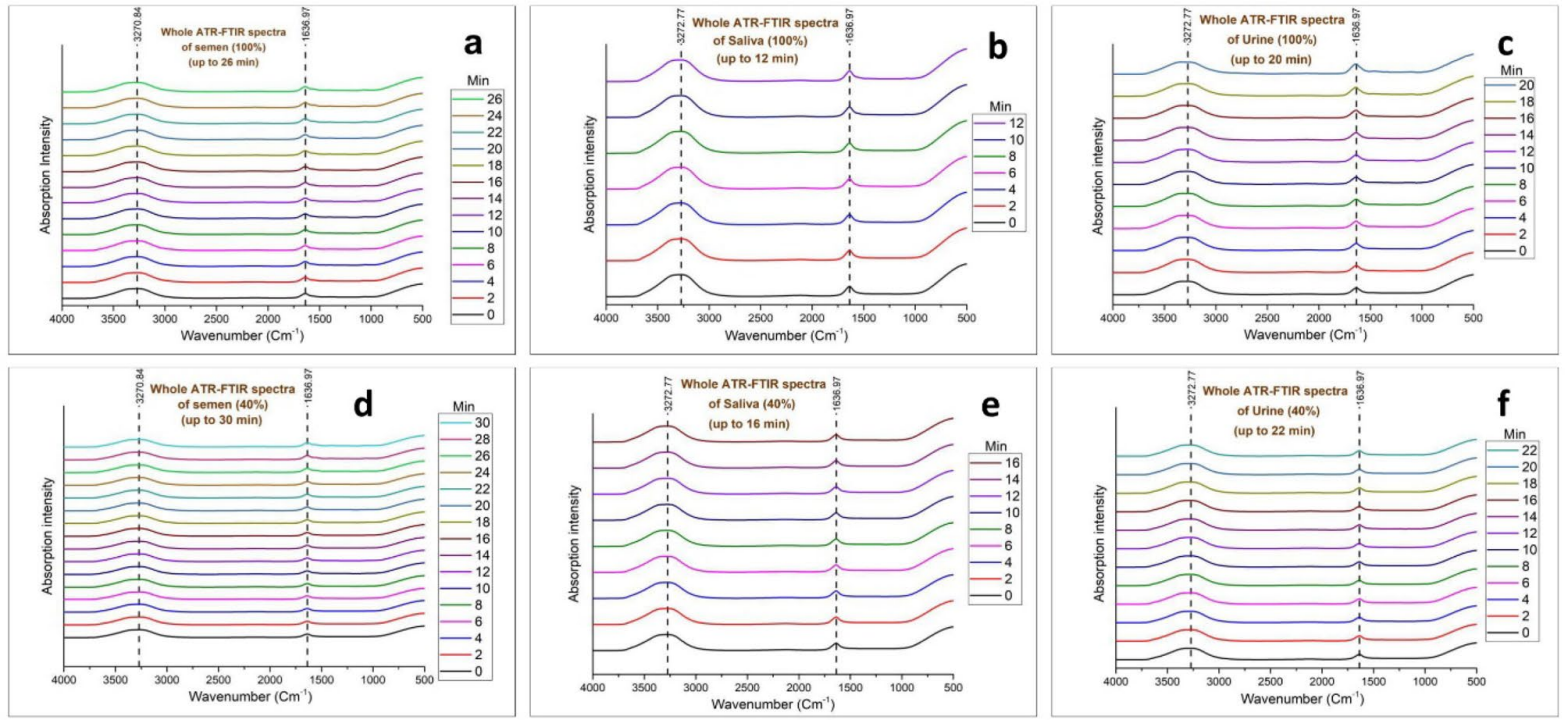
Whole ATR FTIR Phase I spectra for (**a**) 100% semen, (**b**) 100% saliva, (**c**) 100% urine, (**d**) 40% semen, (**e**) 40% saliva, (**f**) 40% urine.

**Figure 2:**
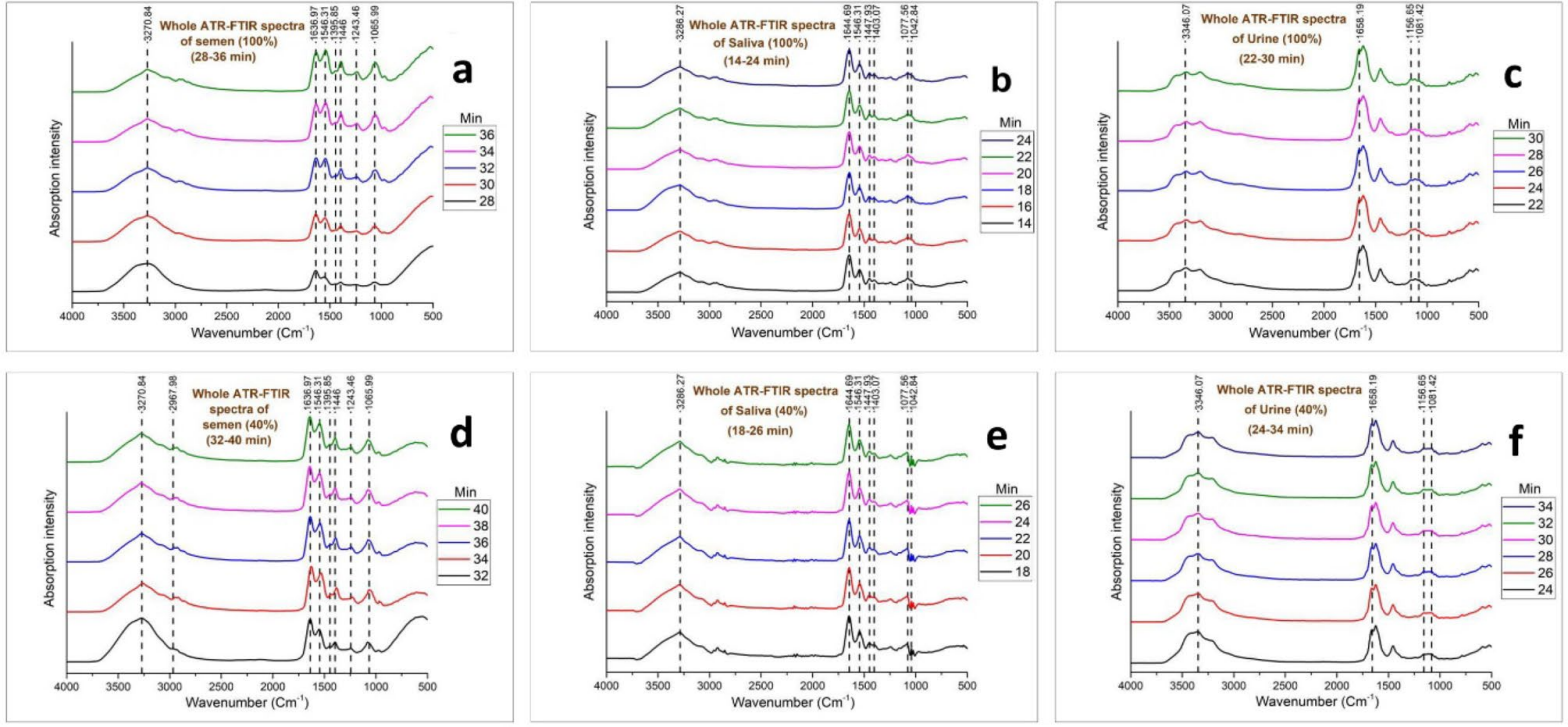
Whole ATR FTIR Phase II spectra for (**a**) 100% semen, (**b**) 100% saliva, (**c**) 100% urine, (**d**) 40% semen, (**e**) 40% saliva, (**f**) 40% urine.

The study was based on three relevant body fluids i.e., semen, saliva, and urine except for blood as a similar study is already reported on it^9^. In this study, the major change is observed in two regions of the spectra i.e., 3300–2800 cm^−1^ (lipids are the major contributors) and 1700–950 cm^−1^ (proteins, nucleic acids, and carbohydrates are the major contributors)^37–39^. The list of peaks identified in the spectra of all three fluids is summarized in Table 1 with their vibrational assignments. All the acquired spectra in the present study showed similarity with previously reported spectra by several researchers. Although, some minor spectral bands are not visible. It would probably occur due to the spectral acquisition timing, as in this study, all the spectra were collected at the earliest stage of the ex-vivo degradation process up to their drying. The minor bands may have occurred at the later stages in the ex-vivo environment^8,39–42^. The spectra in other reported articles were collected at least 4–6 h from the time of deposition^39,40^. Although several peaks were identified in the spectra of each fluid during the study, only a few (age-linked peaks) showed linear changes in their absorption intensity with time. At the initial stage, a similar phenomenon has been observed in the drying of every body fluid. For the first several minutes, only two strong absorption peaks were visible (Fig. 1a–f). These two peaks at 3270–3273 cm^−1^ (O–H stretching) and 1637 cm^−1^ (approx.) (scissoring of two H atoms bonded with O molecule) appeared due to the high amount of water in all the fresh fluid samples^43–45^. Similar results were obtained in the study by Zhang et al., on blood^9^. After a certain amount of time multiple significant peaks corresponding to the biochemical profile of the fluids are revealed throughout the fingerprint region of the IR spectra (Fig. 2a–f). Following the trend in each fluid, the whole drying time was divided into two phases.

**Table 1:** Strong, medium and weak peaks observed in all the three body fluids during their ATR-FTIR spectral analysis with their vibrational modes and spectral assignments.

Body fluid	Wave number (cm^−1^) (approx.)	Spectral assignment
Semen	3271	O–H stretching (Phase I) and Symmetric N–H stretching of Amide A (Phase II)^37,38,39^
	1637	H–O–H scissoring (Phase I) and C=O stretching (Amide I)^37,38,39^
	***2968***	Asymmetric CH_3_ stretching^37,38,39^
	***1546***	N–H bending and C–N stretching (Amide II)^37,38,39^
	*1446*	C–H bending of CH_2_ and CH_3_^37,38,39,41^
	***1396***	C=O stretching in COO^−^ (Fatty acids and polysaccharides)^39^
	1317 and ***1243***	N–H bending and C–N stretching (Amide III)^37,39^
	***1066*** and 970	CH_2_OH groups, C–O stretching and COH groups, symmetric Glycosylated proteins, PO_2_—stretching (Glycosylated proteins)^37,38,39^
*Saliva*	**3273 and 3286**	O–H stretching (Phase I) and Symmetric N–H stretching of Amide A (Phase II)^37,38,39^
	3076	Amide B^37,46,39^
	2958	Asymmetric CH_3_ stretching^37,38,39^
	2881	Asymmetric CH_2_ (methylene) stretching^38,39,[Bibr CR40][Bibr CR42]^
	**1637 and 1645**	H–O-H scissoring (Phase I) and C=O stretching (Amide I)^38,42^
	***1546***	N–H bending and C-N stretching (Amide II)^38,39,46^
	***1448***	C–H bending of CH_2_ and CH_3_^39,40,42,47^
	***1404***	Symmetric CH^42,47^
	1317 and 1243	N–H bending and C–N stretching (Amide III)^37,39, 47^
	***1078*** and ***1043***	CH_2_OH groups, C–O stretching and COH groups, symmetric Glycosylated proteins, PO_2_—stretching (Glycosylated proteins)^37–39^
Urine	***3346***	Asymmetric stretching of H–O–H^39,40^
	***3273***	O–H stretching (Phase I) and Symmetric N–H stretching of Amide A (Phase II)^37,38,46^
	**3205**	O–H stretching^38–40^
	***1658***; **1637** and **1623**	H–O–H scissoring (Phase I) and C=O stretching (Amide I)^37–39^
	1456	Asymmetric C–N stretching (Urea)^38–40^
	***1157***	NH_2_ deformation (Urea)^39,40^
	***1081***	CH_2_OH groups, C–O stretching and COH groups, symmetric Glycosylated proteins, PO_2_—stretching (Glycosylated proteins)^37–39^

‘Bold’ marks denote the peaks changed during the transition from phase I to phase II and ‘Bold’ + ‘Italic’ mark denotes the age-linked peaks.

Except for the concentrated samples of fluids, diluted samples of 2:3 ratio (40%) were also prepared to investigate the changes in the drying of the fluids. The dilution was kept constant for all the fluids and a similar extended drying time had been recorded. Multiple dilutions can also alter the drying time as these factors can be studied in future studies on these fluids separately with other factors as experimented by Zhang et al., on blood^9^. The dilution of the fluids showed an extended (2–4 min) phase I due to the excess amount of water in the diluted sample. The longest phase I observed in the Semen samples and the shortest in the saliva samples. Phase II was relatively similar in the spectra of both raw and diluted samples. The duration of phase II of three body fluids was relatively the same (10–12 min). Table 2 demonstrates the minimum, maximum, and mean values of both phases. The difference in the drying time of all three fluids is potentially a result of the qualitative and quantitative variability in their biochemical components.

**Table 2:** Summarized the minimum, maximum and mean values of two phases of body fluid drying.

Fluids	Concentration (%)	Phase I (min)			Phase II (min)			Total (min)		
		Max	Min	Mean	Max	Min	Mean	Max	Min	Mean
Semen	100	28	22	26	14	10	10	42	32	36
	40	34	28	30	12	8	10	46	34	40
Saliva	100	14	10	12	14	10	12	28	20	24
	40	20	14	16	12	8	10	32	22	26
Urine	100	24	18	20	12	6	10	36	24	30
	40	26	18	22	14	8	12	40	26	34

**Figure 3:**
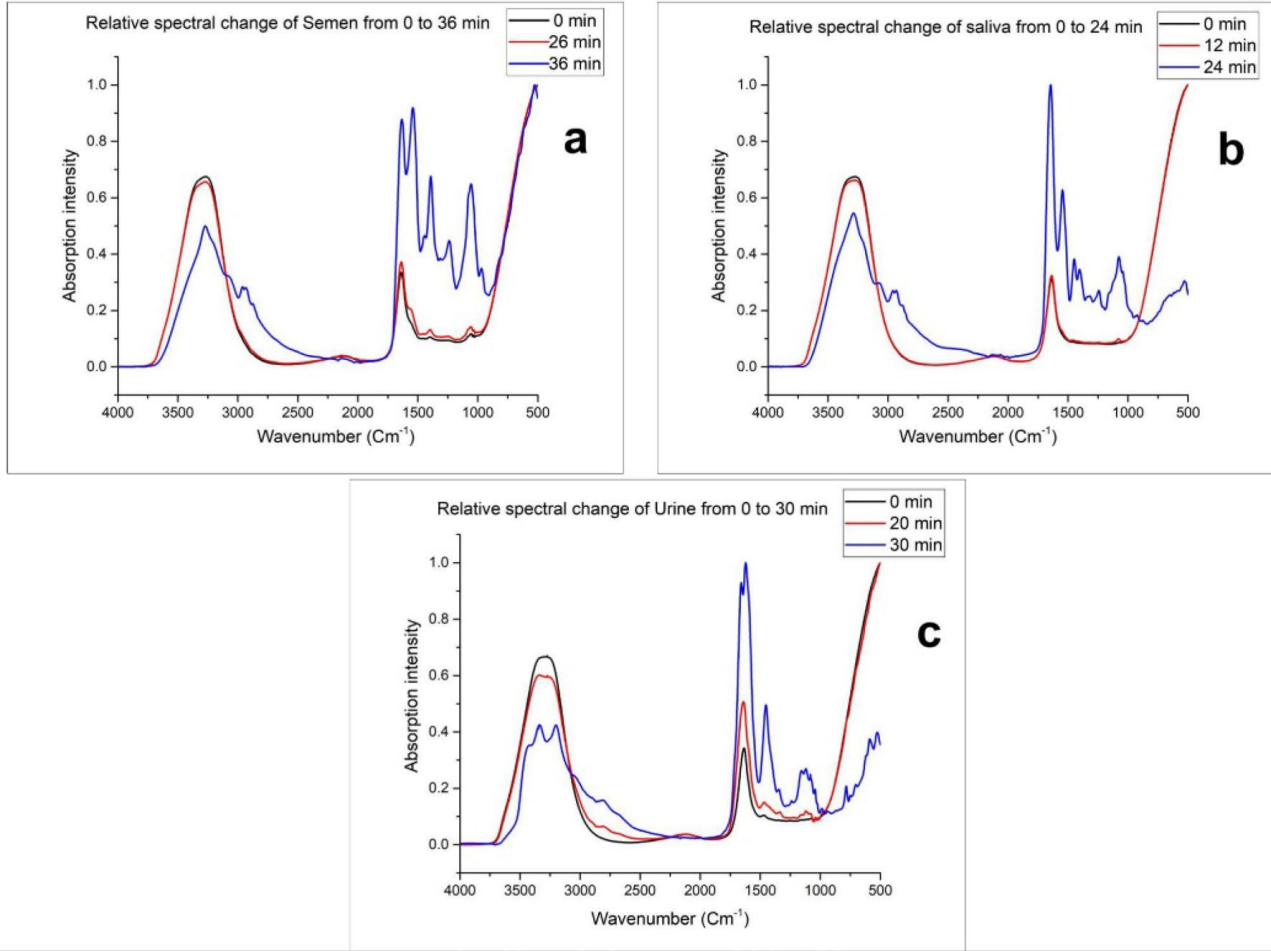
Relative changes in the ATR-FTIR spectra of (**a**) semen, (**b**) saliva and (**c**) urine until drying.

Few researchers reported the correlation between the evaporation of distilled water and time^48,49^. Except for similar height, the peak corresponding to O–H stretching was broader than the peak due to H–O–H scissoring. Except for urine, the absorption intensity of these two peaks showed insignificant change throughout phase I in the spectra of semen and saliva (Figs. 1a–f, 3). On the contrary, Zhang et al.^9^ found a different result for blood as the peak at 3308 cm^−1^ showed very weak but linear absorption change during the early stage. Only the spectra of urine (100% and 40%), showed analogous results with the study by Zhang et al.^9^ as the peak at 3273 cm^−1^ showed a significant decline in the mean absorbance with time during phase I (Figs. 2c,f, 3c).

Phase II is the fast declination stage where a significant amount of water evaporates rapidly and reveals the other peaks and their intensity changes with time in each body fluid. The peaks at 3271 cm^−1^ (Amide A) and 1637 cm^−1^ (Amide I) showed no shift during the whole drying (phases I and II) process of semen stain but the former one sharpens with time and rapidly declined during phase II (Figs. 1a,d, 2a,d) as the peak in Phase II appeared due to the N–H stretching of Amide instead of O–H stretching of water^8,43–45^. In the phase II drying of semen droplets, one strong (1546 cm^−1^: Amide II), two medium (1446 cm^−1^: methylene; CH_2_ and CH_3_ and 1066 cm^−1^: Glycosylated proteins: probably prostate-specific antigen) and three weak (2968 cm^−1^: CH_3_ stretching, 1396 cm^−1^: fatty acids and polysaccharides and 1243 cm^−1^: Amide III) significant age-linked peaks were observed (Fig. 2a,d). Zha et al.^8^ investigated the changes in few similar peaks at marginally different positions i.e., 1539 cm^−1^ (Amide II), 1448 cm^−1^ (Methylene: CH_2_ and CH_3_), 1392 cm^−1^ (Fatty acids and polysaccharides), 1059 cm^−1^ (Prostate-specific antigen). Few more researchers reported the IR spectra of semen in several body fluid identification research articles^38–40^. Phase II spectra of saliva showed the shift of strong peaks at 3273 cm^−1^ (O–H stretching) to 3286 cm^−1^ (amide A) and 1637 cm^−1^ (H–O–H scissoring) to 1645 cm^−1^ (amide I) that indicated the initiation of this phase (Fig. 2b,e). The shifted peak of amide A sharpens following the trend of semen samples. Among others, one strong and sharp (1546 cm^−1^: amide II), four weak (1448 and 1403 cm^−1^: Methylene, 1078 cm^−1^, and 1043 cm^−1^: glycosylated proteins) significant age-linked peaks were found (Fig. 2b,e). Including the peak corresponding to amide II, two peaks at 1448 cm^−1^ and 1078 cm^−1^ are similar to the peaks at 1446 and 1066 cm^−1^ in the spectra of semen and placed at marginally different positions. But the intensity of the peak corresponding to glycosylated protein is relatively weaker in the spectra of saliva. The peaks in phase I spectra of urine bifurcated in phase II. The peak at 3273 cm^−1^ divided into 3346 cm^−1^ and 3205 cm^−1^ and 1637 cm^−1^ divided into 1623 and 1658 cm^−1^ (Fig. 2c,f). One strong (1658 cm^−1^: amide I), one medium (3346 cm^−1^: H–O-H stretching), and 2 weak (1156 cm^−1^: urea and 1081 cm^−1^: Glycosylated proteins) peaks were observed in the phase II spectra of urine samples (Fig. 2c,f) that significantly changes during the drying process. The peak at 1081 cm^−1^ is similar to the peaks at 1066 cm^−1^ and 1078 cm^−1^ of semen and saliva, respectively. Due to the presence of a significant quantity of prostate-specific antigens in semen, the peak corresponding to glycosylated protein is stronger in its spectra than saliva and urine^37–39^. In several previous literatures on the IR signature of saliva and urine, the above-mentioned peaks were reported by researchers^37–42^. Amide A, I, II, and glycosylated proteins are the common biochemical components found in all the 3 body fluids. Elkins, Orphanou, and Takamura et al.^37–39^ previously reported the presence of the common biochemicals in all these body fluids in their article on body fluid identification by ATR-FTIR. In Phase II, all the age-linked peaks of each fluid showed a linear relationship between the mean absorbance at each time point and TSD (Fig. 2a–f).

### Statistical results

The regression equation of a line is the representation of a prediction model. Table 3 depicts the slopes and intercepts calculated for all the age-linked peaks of three body fluids with a 95% level of significance.

**Table 3:** Slopes and intercepts of age-linked peaks of (a) semen (b) saliva (c) urine at 95% level of significance during the phase II drying.

(a) Peak	Semen (100%)			Semen (40%)		
	Slopes (a.u/min)	Intercepts (a.u)	Significance (95%) (p value)	Slopes (a.u/min)	Intercepts (a.u)	Significance (95%) (p value)
2967.98	0.017	− 0.32	< 0.05	0.004	0.17	< 0.05
1546.31	0.076	− 1.77	< 0.05	0.021	0.07	< 0.05
1446	0.039	− 0.90	< 0.05	0.007	0.12	< 0.05
1395.85	0.072	− 1.78	< 0.05	0.015	− 0.04	< 0.05
1243.46	0.038	− 0.90	< 0.05	0.006	0.11	< 0.05
1065.99	0.055	− 1.32	< 0.05	0.015	− 0.08	< 0.05

(b) Peak	Saliva (100%)			Saliva (40%)		
1546.31	− 0.002	0.65	< 0.05	− 0.012	1.02	< 0.05
1447.93	− 0.002	0.40	< 0.05	− 0.012	0.74	< 0.05
1403.07	− 0.003	0.38	< 0.05	− 0.012	0.69	< 0.05
1077.56	− 0.002	0.42	< 0.05	− 0.011	0.69	< 0.05
1042.84	− 0.003	0.37	< 0.05	− 0.010	0.54	< 0.05

(c) Peak	Urine (100%)			Urine (40%)		
3346.07	− 0.011	0.72	< 0.05	− 0.014	1.14	< 0.05
1658.19	0.003	0.83	< 0.05	0.002	0.85	< 0.05
1156.65	0.003	0.18	< 0.05	0.002	0.20	< 0.05
1081.42	0.003	0.16	< 0.05	0.001	0.22	< 0.05

P value is the measure of the statistical significance of the observed difference. In this table it indicates the significance of the slope and intercepts, which are less than 0.05 at 95% level of significance.

**Figure 4:**
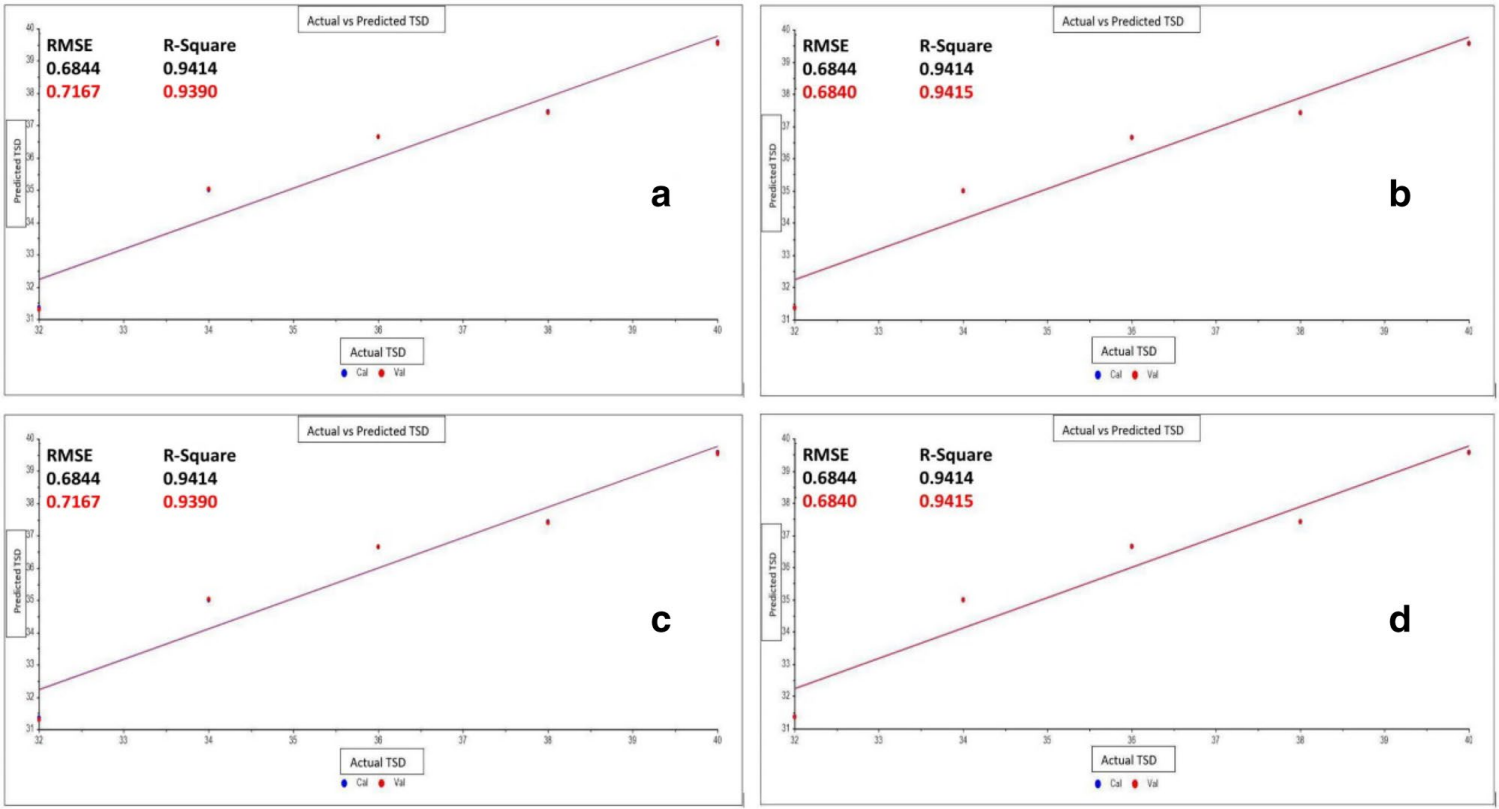
PCR plots of (**a**) actual vs cross validated prediction of TSD for semen (40%), (**b**) actual vs externally validated prediction of TSD for semen (40%), (**c**) actual vs cross validated prediction of TSD for saliva (100%), (**d**) actual vs externally validated prediction of TSD for saliva (100%).

**Figure 5:**
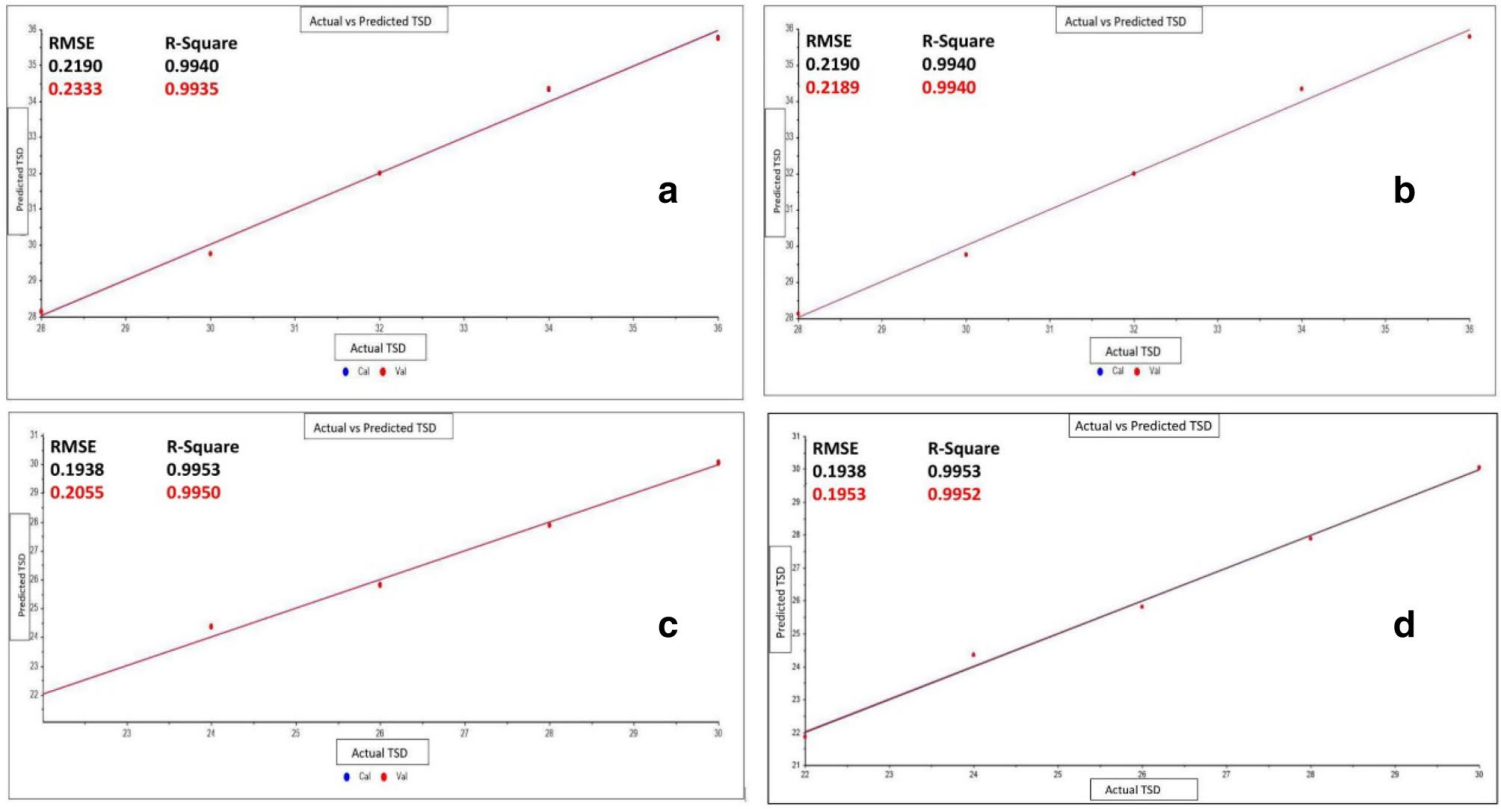
PLSR plots of (**a**) actual vs cross validated prediction of TSD for semen (100%), (**b**) actual vs externally validated prediction of TSD for semen (100%), (**c**) actual vs cross validated prediction of TSD for urine (100%), (**d**) actual vs externally validated prediction of TSD for urine (100%).

Body fluids show significant ex-vivo degradation when exposed for a relatively longer period. While early changes are very limited as only a few relevant peaks due to aging are visible. Hence, the TSD estimation was performed only on the age-linked peaks of three body fluids. Among several strong and medium peaks 6 (semen), 5 (saliva), and 4 (urine) peaks were selected to calculate the TSD of the body fluids. Irrespective of the variation in the numbers of age-linked peaks for the three body fluids, it was evident that the regression models successfully estimated the TSD with very high accuracy. Both Principal Component Regression (PCR) Analysis and Partial Least Square Regression (PLSR) are strong chemometric tools for the estimation of TSD of body fluids as reported in previous studies^1,4–11^. The calculated *R*^2^ values for the calibration and prediction of both models are more than 0.9. While the Root Mean Square Error of Cross-Validation and Prediction (RMSECV and RMSEP) values in both the models for all the three fluids except diluted urine, showed low values (Table 4a,b). Although the initial changes in the IR spectra of body fluids are relatively less distinguishable in comparison to the samples exposed for a longer period, the High *R*^2^ and low RMSE values indicate a good prediction of TSD during this timeframe. In diluted urine samples, the RMSECV (PCRA: 0.7659; PLSR: 0.7622) and RMSEP (PCRA: 0.7383; PLSR: 0.7327) (Table 4a,b) in both the models are relatively higher, that potentially interfere in the accurate age estimation. While, diluted semen also showed higher RMSECV (0.7167) and RMSEP (0.6840) in the PCRA regression model, while PLSR predicts the age for the same condition with significantly higher accuracy with RMSECV and RMSEP values of 0.1897 and 0.1546, (Table 4a,b) respectively. The lower RMSE value also depicts that there is very minute inter-donor variation and low standard deviation values (0.00002–0.00004) of the spectral data obtained from the repeated sampling showed minimal intra-donor variation. Additionally, the RPD (Residual Predictive Deviation) values for each model were also calculated and it has been found that all the values are above 3 which indicates an excellent prediction model accuracy (Fig. 4)^50,51^. Comparatively, PLSR showed better efficiency of prediction than PCRA as for every fluid it records relatively higher *R*^2^ and lower RMSE values than in PCRA. Figures 4 and 5 depicts the PCR and PLS plots of actual vs predicted regression lines for age-linked peaks of three body fluids. Finally, one-way ANOVA has also been applied to the age-linked peaks separately. The F-statistic for all the age-linked peaks showed a significant difference between the time intervals (Table 5).

**Table 4:** (a) Validation results of PCR models for multiple age-linked peaks of three body fluids, (b) validation results of PLSR models for multiple age-linked peaks of three body fluids.

Fluids	Cross-validation		External Prediction		RPD
	*R*^2^	RMSECV (min)	*R*^2^	RMSEP (min)	
**(a) Principal component regression (PCR) analysis table**					
Semen (100%)	0.9935	0.2346	0.9939	0.2201	13.50
Semen (40%)	0.9390	0.7167	0.9415	0.6840	20.53
Saliva (100%)	0.9885	0.3742	0.9891	0.3568	9.64
Saliva (40%)	0.9973	0.1517	0.9973	0.1458	19.53
Urine (100%)	0.9948	0.2084	0.9951	0.1983	15.01
Urine (40%)	0.9518	0.7659	0.9532	0.7383	4.73
**(b) Partial least square regression (PLSR) analysis table**					
Semen (100%)	0.9935	0.2333	0.9940	0.2189	13.57
Semen (40%)	0.9957	0.1897	0.9970	0.1546	19.26
Saliva (100%)	0.9889	0.3737	0.9891	0.3561	9.67
Saliva (40%)	0.9969	0.1618	0.9973	0.1457	20.44
Urine (100%)	0.9950	0.2055	0.9952	0.1953	15.24
Urine (40%)	0.9523	0.7622	0.9540	0.7327	4.78

**Table 5:** Calculated F-statistics by one way ANOVA for all the age-linked peaks of three body fluids.

Fluid	F-statistic (95% level of significance)					
Semen (100%)	2968 cm^−1^	1546 cm^−1^	1446 cm^−1^	1396 cm^−1^	1243 cm^−1^	1066 cm^−1^
	2.87	5.44	1.09	2.83	1.26	2.58
Semen (40%)	1.11	6.68	6.28	2.82	3.83	3.17
Saliva (100%)	1546 cm^−1^	1448 cm^−1^	1404 cm^−1^	1078 cm^−1^	1043 cm^−1^	
	4.72	6.57	8.80	5.24	9.56	
Saliva (40%)	1.65	1.37	1.46	9.33	7.47	
Urine (100%)	3346 cm^−1^	1658 cm^−1^	1156 cm^−1^	1081 cm^−1^		
	8.84	1.04	6.84	1.21		
Urine (40%)	2.89	3.95	2.59	1.61		

In the present study three forensically significant body fluids other than blood i.e., semen, saliva, and urine were considered to explore the instantaneous changes in their ATR-FTIR spectra up to their drying. This study revealed that all the body fluids undergo significant water loss at the initial stage of their ex-vivo degradation. This rapid loss of water significantly divided the drying process into two phases as the first phase consists of slow evaporation with minimal spectral change with a major contribution of water and the second phase depicts relatively faster evaporation. These two phases can be distinguishable from the ATR-FTIR spectra of each fluid which is a significant marker for estimating the time since deposition of the fluid(s). This study also revealed the spectral regions of interest for the TSD estimation of these fluids as saliva and urine are not explored previously for this purpose. Additionally, if a body fluid is accidentally diluted during the deposition on a wet nonporous substrate during the earliest phase post-deposition, the accuracy of the estimation of TSD can be significantly altered as the water evaporation potentially take more time. In the practical scenario, fresh body fluid samples in liquid and semi-liquid (fluid samples with loss of a certain quantity of water since deposition) conditions from any non-porous surface (e.g., glass, metal, tile, etc.) can be easily collected through a pipette-like apparatus. We can acquire the spectra of the freshly collected fluid with a portable IR instrument containing an ATR-FTIR crystal face exclusively dedicated to forensic crime scene investigation purposes. Although the drying process of body fluids is different on porous substrates like, cloth fabrics, carpets, etc. Hence, further experiments on this subject can be framed based on several factors like, different concentrations, quantities, and interference of porous substrates. Despite, relatively low changes in the IR absorption, chemometric tools like; PCR and PLSR successfully estimate the TSD for each fluid during the initial spectral changes with a very low RMSECV and high *R*^2^ values. Hence, the results (spectral and statistical) of this study potentially be used as a reference for the further TSD studies on these three fluids with a longer timeframe including different factors.

## Materials and methods

### Sampling

Samples of saliva, semen, and urine were collected from randomly selected eight healthy volunteers (28–40 years). All the methods of this study were carried out in accordance with the World Medical Association Declaration of Helsinki. The experimental protocols were approved by the Ethical Committee of Maharani Laxmi Bai Medical College (4647/IEC/2020/SC-1), Bundelkhand University, Jhansi, Uttar Pradesh, India. All the donors were informed about the nature and procedure of the work and written informed consent was taken from each donor. Each body fluid from a donor was collected separately in a glass test tube(s) just before the spectral analysis to avoid any loss of time after its release from the human body. All the fluids were collected by voluntary secretion, ejaculation, and excretion process without using any invasive technique. Spectra of fluids were obtained in two different concentrations i.e., 100% and 40% (2:3) to investigate the effect of water content on the drying time. The dilution concentration was randomly selected for this study to investigate the difference in drying time between concentrated and dilute fluid samples. 40% solutions of all the fluids were prepared by mixing 2 parts of the fluid and 3 parts of distilled water in a test tube instantly after the collection of the fluid. For experimental purposes, saliva and urine samples were repeatedly taken for three consecutive days, and semen was collected three times with an interval of 3 days to observe any intra-variations of their composition. All the repeated samples were collected from the same individuals.

### Collection and pretreatment of FTIR spectra

IR spectra of all the body fluid samples were collected by a ‘Spectrum Two’ FTIR spectrophotometer manufactured by Perkin Elmer corporation, equipped with a 2 mm diameter diamond crystal ATR accessory and spectrum software (Version 10.0). The spectrum software is used for the collection of spectra. The crystal face was cleaned with a 70% methanol solution before drop the fluid sample for each spectral measurement. One drop (approximately 50 µL) of each body fluid was separately added onto the crystal from a constant height of 4 cm. The crystal was used as the drying surface to reduce any loss of water from the fluids during the transfer of the samples from the substrates. Throughout the whole study, the volumes of fluids were kept relatively constant. The experiment was performed in the month of January. The approximate temperature and relative humidity during the complete spectral collection varied between 13 and 17 °C and 56–75%, respectively. As the ambient conditions during the study did not vary significantly, the effect of variable temperature and humidity were not considered in this study. The spectra of each fluid were collected with an interval of two minutes immediately after placing the droplet on the crystal within the range of 4000 cm^−1^ to 500 cm^−1^ with 12 scans and a resolution of 4/cm. In this study, the term ‘drying out’ is the time point at which there was a negligible change between the three consecutive scans taken at a 2-min interval^26^. At this point, the droplet transformed into a dried stain. Before analyzing the obtained data, all the spectra of body fluids were preprocessed by using Unscrambler X software with several spectral corrections i.e., baseline offset, spectral smoothing with Savitzky-Golay algorithm including 13 smoothing points and 3 polynomial orders in a symmetric kernel and range normalization^52,53^.

### Statistical and chemometric analysis

The mean values of the absorbance of all the significant peaks and the drying time were calculated. Peak identification, fitting, and statistical analysis were carried out by using Origin Pro 2016 and Microsoft excel 2019 software. The correlation coefficient (*R*) and coefficient of determination (*R*^2^) between the TSD and changes in the absorbance values for each body fluid was established with a fitting correlation equation. All the equations showed a ‘*P*’ value of < 0.05 which is statistically significant. PCR and PLSR are the two most frequently used tools for prediction model creation and estimation studies. Both of these tools decompose the multiple X variables with respect to the values of Y variables and generate single values for each sample and establish a correlation between X and Y.1 In the present study, the time has been considered as Y and the different age-linked peaks with absorbance values are considered as X. All the chemometric analyses were performed by using ‘Unscrambler X’ (CAMO Analytics) software. RMSECV and RMSEP were calculated to check the consistency and predictive ability of the regression model. Higher *R*^2^ values and lower RMSE values are indicative of a good prediction model. Full cross-validation was performed for each fluid. Eight samples randomly for each fluid were selected for the model creation and two randomly left out samples were applied for external validation purposes.

## Data Availability

All data generated or analysed during this study are available from the corresponding author on reasonable request.
